# Computed tomography window affects kidney stones measurements

**DOI:** 10.1590/S1677-5538.IBJU.2018.0819

**Published:** 2019-01-29

**Authors:** Alexandre Danilovic, Bruno Aragão Rocha, Giovanni Scala Marchini, Olivier Traxer, Carlos Batagello, Fabio Carvalho Vicentini, Fábio César Miranda Torricelli, Miguel Srougi, William Carlos Nahas, Eduardo Mazzucchi

**Affiliations:** 1 Hospital das Clínicas Faculdade de Medicina Universidade de São Paulo SP Brasil Departamento de Urologia do Hospital das Clínicas da Faculdade de Medicina da Universidade de São Paulo, SP, Brasil;; 2 Faculdade de Medicina Universidade de São Paulo SP Brasil Departamento de Radiologia do Hospital das Clínicas da Faculdade de Medicina da Universidade de São Paulo, SP, Brasil;; 3 Sorbonne Université Hôpital Tenon Paris France Sorbonne Université, GRC n 20 Lithiase Renale, AP-HP, Hôpital Tenon, F-75020 Paris, France. University, Paris, France

**Keywords:** Kidney Calculi, Nephrolithiasis, Patient Outcome Assessment

## Abstract

**Objectives:**

Measurements of stone features may vary according to the non-contrast computed tomography (NCCT) technique. Using magnified bone window is the most accurate method to measure urinary stones. Possible differences between stone measurements in different NCCT windows have not been evaluated in stones located in the kidney. The aim of this study is to compare measurements of kidney stone features between NCCT bone and soft tissue windows in patients submitted to retrograde intrarenal surgery (RIRS).

**Materials and Methods:**

Preoperative and 90th postoperative day NCCT were performed in 92 consecutive symptomatic adult patients (115 renal units) with kidney stones between 5 mm to 20 mm (< 15 mm in the lower calyx) treated by RIRS. NCCT were evaluated in the magnified bone window and soft tissue window in three axes in a different time by a single radiologist blinded for the measurements of the NCCT other method.

**Results:**

Stone largest size (7.92±3.81 vs. 9.13±4.08; mm), volume (435.5±472.7 vs. 683.1±665.0; mm3) and density (989.4±330.2 vs. 893.0±324.6; HU) differed between bone and soft-tissue windows, respectively (p<0.0001) 5.2% of the renal units (6/115) were reclassified from residual fragments > 2 mm on soft tissue window to 0-2 mm on bone window.

**Conclusion:**

Kidney stone measurements vary according to NCCT window. Measurements in soft tissue window NCCT of stone diameter and volume are larger and stone density is lesser than in bone window. These differences may have impact on clinical decisions.

## INTRODUCTION

Non-contrast computed tomography (NCCT) has become the gold standard for diagnosing urinary stones (1). NCCT is able to provide stone features as size, volume and density that are relevant for making clinical decisions. Stone size is of paramount importance for spontaneous stone passage (2). Stone volume is the best predictor of operative time and is an independent predictor of stone-free status in retrograde intrarenal surgery (RIRS) for kidney stones (3, 4). Hounsfield units (HU) density is able to differentiate uric acid stones, to predict success of shockwave lithotripsy and to impact on operative time of RIRS using holmium laser lithotripsy (5-9). However, the measurements of these stone features may vary according to the NCCT technique (10).

Most data previously reported about urinary stones features were measured in soft tissue window conventional-dose NCCT (3-9). However, it has been demonstrated in distal ureteral stones that magnified soft tissue window NCCT is a poor predictor of the largest stone dimension (11) and that magnified bone window is the most accurate method to measure urinary stones in vitro and in vivo (12). The possible differences between windows of NCCT have not been evaluated in stones located in the kidney. The aim of this study is to compare kidney stone features between bone and soft tissue windows using the currently best practice protocol NCCT in patients submitted to RIRS.

## MATERIALS AND METHODS

From August 2016 to August 2017, preoperative and 90th postoperative day (POD) NCCT were performed in consecutive symptomatic adult patients with kidney stones that chose to be treated by RIRS.

RIRS was offered as an option for the treatment of symptomatic kidney stones between 5mm to 20mm. We limited the option of RIRS in the lower calyx for stones up to 15mm in an attempt to maximize stone free rate and to reduce flexible ureteroscope damage (13-15). Lower calyx stones larger than 15mm were treated by percutaneous nephrolithotomy (16).

Patients with kidney malformations, ureteral stenosis, previous ipsilateral endoscopic or open kidney surgery, hydronephrosis, indwelling double J stent and contraindications for RIRS were excluded.

NCCT was performed using a 64-slice GE Lightspeed CT Scanner® (General Eletric®, USA) with a slice thickness of 1mm and radiation low-dose protocol (low tube charge current-60mAs) in patients with Body Mass Index-BMI <30Kg/m^2^ and conventional protocol (160mAs) in patients with >30Kg/m^2^. Low-dose NCCT is recommended for the evaluation of urinary stones in non-obese patients due to equivalent detection of urolithiasis and stone measurements comparing to conventional dose NCCT using less ionized radiation (17, 18).

**Figure 1:**
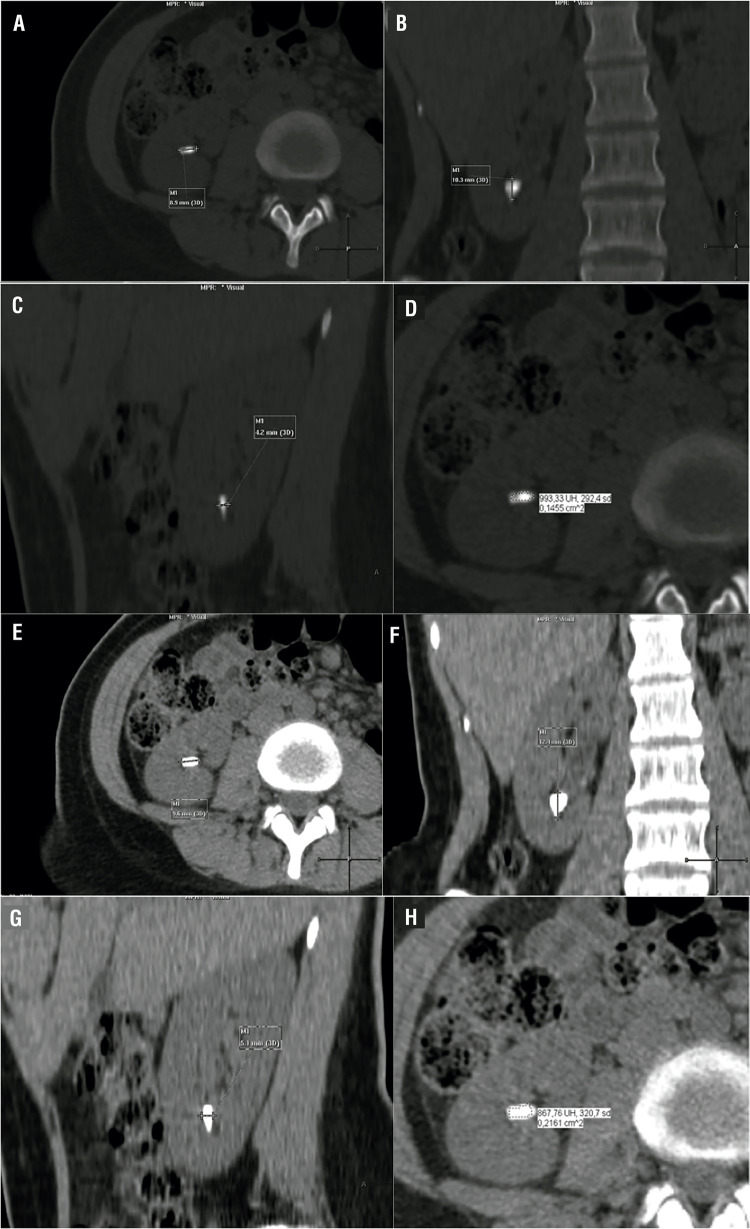
Preoperative magnified (400%) NCCT bone window (ww 1600HU/wl 500HU) vs. soft tissue window (ww 400HU/wl 40HU). A) bone window axial stone diameter, B) bone window coronal stone diameter, C) bone window sagittal stone diameter, D) bone window stone density, E) soft tissue window axial stone diameter, F) soft tissue window coronal stone diameter, G) soft tissue window sagittal diameter, H) soft tissue window stone density.

**Figure 2:**
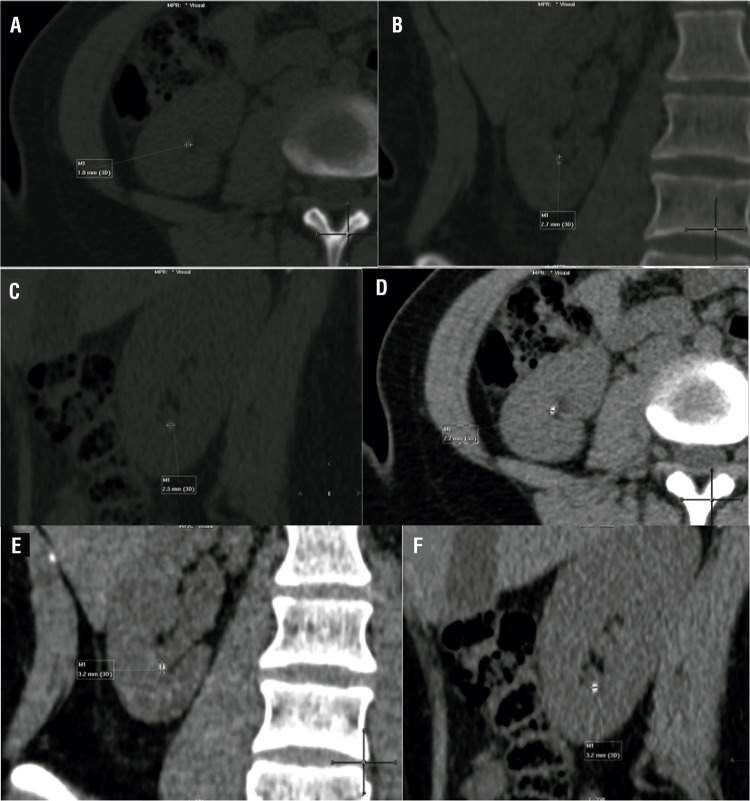
Postoperative magnified (400%) NCCT bone window (ww 1600HU/wl 500HU) vs. soft tissue window (ww 400HU/wl 40HU). A) bone window axial stone diameter, B) bone window coronal stone diameter, C) bone window sagittal stone diameter, D) soft tissue window axial stone diameter, E) soft tissue window coronal stone diameter, F) soft tissue window sagittal diameter.

Magnified (400%) NCCT were evaluated first in bone window (width, 1600HU/level, 500HU) in axial, coronal and sagittal plane and then NCCT were evaluated in soft tissue window (width, 400HU/level, 40HU) by the same radiologist blinded for the results of the measurements of the bone window NCCT (Figure-1). Postoperative NCCT stone measurements were performed in the same fashion (Figure-2).

Stone density was measured by free hand ROI determination coincident with the stone borders. Stone volume was calculated as length x width x depth x π x 0.167 (1, 19). Residual fragments were categorized as 0 when no residual fragments exists, 0-2mm and >2mm.

## STATISTICAL ANALYSIS

Bone and soft tissue window NCCT results were compared using paired T-test, Wilcoxon Signed Rank. Bone and soft tissue window NCCT residual fragments were compared using McNemar-Bowker test of symmetry. Sample size was calculated based on the percentage of renal units with residual fragments more than 2mm by NCCT of 38% (20). Therefore, the sample size for a bicaudal test with significance level of 5% and test power of 95% is 115 renal units.

SAS 9.0 program® (SAS Institute Inc., Cary, NC, USA) was used with a significance level of 5%.

## RESULTS

Ninety-two patients were successfully submitted to RIRS. Bilateral procedures were performed in 23 patients (25%) resulting in 115 renal units operated. Body Mass Index was 28.1±4.8, 19.0-45.5Kg/m^2^ (mean±SD, range). Twenty-eight patients (35 renal units, 30.4%) were obese (BMI >30Kg/m^2^) and were submitted to conventional-dose NCCT. Stone features evaluated by bone and soft tissue windows are compared in Table-1. Stone largest size, volume and density differed between the two methods (p <0.0001). Although residual fragments diameter was not significantly different when evaluated by NCCT using bone or soft tissue window (p=0.1116) (Table-2), 5.2% of the renal units (6/115) were reclassified from residual fragments >2mm to 0-2mm.

**Table 1:** Comparison between pre-operative NCCT bone and soft tissue windows of the stone features of the115 renal units submitted to RIRS.

Stone features	Bone window	Soft Tissue window	p-value
Multiple stones (%)	69 (60.0)	69 (60.0)	1.000
Stone size (mean±SD, mm)	7.92±3.81	9.13±4.08	<0.0001
Stone volume (mean±SD, mm^3^)	435.5±472.7	683.1±665.0	<0.0001
Stone density (mean±SD, HU)	989.4±330.2	893.0±324.6	<0.0001

**Table 2:** Comparison between post-operative NCCT bone and soft tissue windows of the residual stone size of the115 renal units.

Residual stone size	Bone window	Soft Tissue window	p-value
0 mm, N (%)	86/115 (74.8)	86/115 (74.8)	0.1116
0-2 mm, N (%)	10/115 (8.7)	4/115 (3.5)	
> 2 mm, N (%)	19/115 (16.5)	25/115 (21.7)	

The 90th postoperative day bone and soft tissue window NCCT revealed one asymptomatic small subcapsular hematoma in a stone free renal unit and two asymptomatic hydronephrosis, one in a stone free renal unit and other in a renal unit with >2mm residual fragment.

## DISCUSSION

Low dose NCCT is the current gold standard for the evaluation of urinary stone disease due to its lower radiation exposure (0.7-2.8mSv) than conventional dose NCCT (8-16mSv) and high pooled sensitivity of 0.966 (95% CI, 0.950-0.978) and a pooled specificity of 0.949 (95% CI, 0.920-0.970), which are equivalent to conventional dose NCCT sensitivity of 97% and specificity of 96% (17, 18). Besides, other authors found no significant difference in the measurement of stone size and HU between low dose and conventional NCCT (17, 21). However, conventional dose NCCT is still recommended for the evaluation of urinary stones in obese patient (BMI >30Kg/m^2^) (1).

We used low-dose NCCT with 60mAs in non-obese patients (BMI <30kg/m^2^) and NCCT with 160mAs in obese patients for this study to minimize the radiation exposure without compromising image quality (22, 23). NCCT image noise varies proportional to the value of the square root of the miliampere product. Higher noise from ultra low-dose NCCT (24) may decrease accuracy in detecting small residual fragments (<3mm) (25).

Other authors stressed the importance of standardization of making measurements on NCCT images (10). They demonstrated a larger variability for inter-reader (±1.3mm) than intra-reader (26). Narayan et al. demonstrated that stone density measurements vary depending on window, plane and ROI technique. They recommend that clinicians select a single ROI measurement technique and remain consistent to minimize variability (27). However, ROI measurement should include the periphery of the stone as we did by free hand technique to better represent the entire nature of that stone. As a result, we may better predict laser and operative time or even which laser technique (dusting, fragmentation or popcorn) is better according to stone density. A single senior radiologist evaluated all NCCT studies, in a different time, blinded for the results of the bone window NCCT stone measurements. This might have reduced the possible measurement bias.

Magnified bone window NCCT should be preferred for urinary stone evaluation due to better image quality for dense objects as it minimizes noise artifacts close to the stone limits (12, 25). In vitro study already demonstrated that soft tissue window overestimates stone size and bone window provides best accuracy (26). Clinically, it was shown that bone window allows a visual distinction between a stent and a stone (28, 29). On the other hand, urologists are more familiar with soft tissue window when looking at NCCT and most data related to stone features and NCCT were produced using soft tissue window.

In order to establish if there is a difference between kidney stone measurements in bone and soft tissue windows, we compared preoperative urinary stone features and 90 POD results in both windows. We demonstrated that preoperative bone window NCCT image produce smaller size and volume stone and bigger density stone than soft tissue window (p <0.0001). These results have major clinical impact because regarding stone treatment, every millimeter counts for the decision to actively treat or not. Besides, stone-free rates of all modalities of active treatment of renal calculi are based on size, burden or volume of stone. We found differences in stone density probably because of variation in positioning their regions-of-interest due to different time of measurement as stressed by Williams Jr. (30) and to less noise in the stone surround.

Although we found no significant differences between both windows in the stone free status and complications in the follow-up evaluation, it is important to notice that in 5.2% of the renal units operated the difference in size of residual fragments was clinically relevant. According to previous studies, residual fragments >2mm are more likely to experience growth and cause disturbance to patients (16, 31-33). Therefore, the correct measurement of residual fragment is of upmost importance to plain reintervention.

Our study has several strengths. It is a prospective study using preoperative and postoperative current best practice NCCT after RIRS in patients without kidney malformations, ureteral stenosis, previous ipsilateral endoscopic or open kidney surgery, hydronephrosis or indwelling double J stent, providing more accurate results. To the best of our knowledge, it is the first study to prospectively address a comparison between NCCT bone and soft tissue windows for kidney stones.

This study has some limitations. We did not compare the real size of the intact stone to the NCCT measured size because the stones were broken during RIRS. However, other authors already proved that bone window is more accurate comparing to real distal ureteral stones (11). Also, we used two different NCCT protocols. We used low-dose NCCT in non-obese patients and conventional dose NCCT in obese patients in order to minimize the radiation exposure. Previous authors showed that low-dose NCCT did not compromise image quality (22, 23). However, we did not examine these subgroups separately. Another limitation is the single center nature of our study. Therefore, our results should be validated by other high volume centers.

## CONCLUSIONS

Kidney stone measurements vary according to NCCT window. Measurements in soft tissue window NCCT of stone diameter and volume are larger and stone density is lesser than in bone window. These differences may have impact on clinical decisions.
